# Expanding congenital Zika syndrome: normocephalic infants with neurological and visual deficits

**DOI:** 10.1097/MS9.0000000000005021

**Published:** 2026-04-21

**Authors:** Asra Amjad, Sadia Tameez-Ud-Din, Fred Segawa

**Affiliations:** a Department of Medicine, Islamic International Medical College, (Riphah International University), Rawalpindi, Pakistan; b Department of Medicine, Foundation University Medical College, Islamabad, Pakistan; c Department of Medicine, Makerere University, College of Health Sciences, Kampala, Central Region, Uganda

*To the Editor*,

The Zika virus is a flavivirus (RNA virus) that is often found at elevations of 2000 m above sea level and is transmitted to people by *Aedes aegypti* and *Aedes albopictus* mosquitoes^[^1^]^. To fully realize the scope of its clinical influence, one must comprehend the dynamics of its transmission. Aedes mosquito bites and vertical transfer from an infected mother to the fetus through the placenta, particularly during the first and second trimesters, are the two modes of transmission. The broad range of disease severity observed in affected populations is consistent with these transmission mechanisms. Most of the time, the infection is not serious, but infection during pregnancy is linked to fetal genetic abnormalities, such as microcephaly and other deformities, and CNS lesions^[^2^]^. Emerging data point to a more comprehensive clinical profile than the traditional manifestation of microcephaly.

Children with congenital Zika virus have neurological problems (e.g., developmental delay, seizures, visual impairments) together with normal head circumference. The developmental outcomes in this patient population are further clarified by recent longitudinal research.

At 2 years of age, prospective monitoring of normocephalic children exposed to ZIKV revealed no significant impairments in cognition, motor abilities, language, or behavior. However, visual acuity, retinal atrophy, macular pigment mottling, and contrast sensitivity deficiencies indicate that the visual system may be particularly susceptible to prenatal ZIKV exposure^[^3^]^. Up to 80% of infected individuals are thought to be asymptomatic during the acute stage of Zika virus infection, which is typically characterized by mild fever, arthralgia, maculopapular rash with pruritis, and conjunctivitis. Even though the infection’s clinical course is often minor, serious consequences can still arise.

Usually, symptoms persist for only a few days after a 12-day incubation period. Guillain–Barré syndrome was linked to infections in healthy individuals in addition to the effects during pregnancy^[^4^]^. Even while these symptoms are usually temporary, the consequences during pregnancy are nonetheless very serious.

Pregnancy-related infections, however, can result in intrauterine growth restriction, birth abnormalities, visual and hearing loss, and cognitive and speech issues in addition to social and physical development issues in the offspring^[^5^]^. Therefore, accurate diagnosis is crucial, yet in endemic areas it remains difficult.

Because the symptoms of the Zika virus are similar to those of dengue fever, the illness is frequently misdiagnosed. The pathophysiological mechanisms at the cellular level shed light on these clinical symptoms.

Neuroglial components are directly infected during early gestation, with secondary effects resulting from related ischemia. Despite the broad range of histology, some cases show significant calcification and destructive lesions with agyria, with neurotropic effects^[^6^]^. These anatomical alterations can explain the neurological and visual impairments seen in afflicted newborns. Given the high rate of occipital cortex volume loss seen in CZS-affected infants, particularly in those with visual impairment and normal eye examinations, the visual impairment is most likely associated with cortical blindness, specifically due to occipital cortex structural abnormalities^[^7^]^. Strong diagnostic techniques are necessary for early detection because of this complexity.

Numerous attempts have been made to identify an accurate, dependable, and reasonably priced diagnostic test. Urine and sperm were offered as alternatives to serum, which was the primary biological material employed in serological diagnostics. Both structural and nonstructural ZIKV genomic sections were employed in molecular techniques. While molecular assays are more specific, serological tests are quicker and less costly. The most suitable and trustworthy approach to obtain accurate diagnostic results is to use both approaches^[^8^]^. Comprehensive clinical evaluation is essential to risk assessment in addition to laboratory tests.

To predict unfavorable fetal outcomes associated with the Zika virus, a complete maternal history that includes exposure to the virus is required, as is the use of prenatal diagnostic technologies, such as cranial ultrasound in the first trimester to assess central nervous system abnormalities and MRI to detect cranial lesions^[^9,10^]^. Rapid and cooperative research in all fields, basic science, diagnostics, epidemiology, clinical medicine, and public health, is required since there are so many unresolved concerns regarding ZIKV and its impact on pregnancy and fetal development. Future research goals include, but are not limited to, improving the identification of cases and outcomes with long-term follow-up of exposed mothers and infants (including cohort and case–control studies with collection of appropriate potential confounder data), developing better diagnostic tools given the severe limitations of RT-PCR and serologic testing, as well as better understanding of current tools (sensitivity, specificity, positive and negative predictive values), understanding other potential modes of transmission, especially breastfeeding and sexual contact, understanding the role of asymptomatic infection (risk of transmission, especially in pregnancy), animal models to definitely prove or disprove causal link between maternal ZIKV infection and fetal brain insult, novel methods of vector control and vaccine and antiviral pharmaceutical development. The necessity to update case definitions to include normocephalic children who may nonetheless acquire neurological abnormalities later in life is one of the significant public health consequences highlighted by the growing understanding of Congenital Zika Syndrome. Under-recognition of cases linked to Zika virus infection has probably resulted from relying solely on microcephaly, highlighting the significance of bolstering monitoring systems to include more comprehensive clinical criteria and long-term developmental follow-up. To ensure early detection, accurate reporting, and prompt action, it is also essential to improve awareness and training among pediatricians and other healthcare professionals. Taken together, these actions will enhance disease surveillance, guide the distribution of resources, and support more successful public health initiatives in affected areas. In conclusion, the identification of normocephalic presentations within Congenital Zika Syndrome demands a more complex, multidisciplinary approach and challenges established diagnostic paradigms. To properly capture the illness burden and lessen its impact on impacted people, it will be crucial to prioritize long-term follow-up, integrate clinical and laboratory diagnosis, and expand monitoring criteria.

This manuscript is in compliant to the TITAN Guidelines, 2025, declaring no use of AI^[^11^]^.

## Data Availability

Not applicable.
